# Neural Processing of Emotional Facial and Semantic Expressions in Euthymic Bipolar Disorder (BD) and Its Association with Theory of Mind (ToM)

**DOI:** 10.1371/journal.pone.0046877

**Published:** 2012-10-08

**Authors:** Agustin Ibanez, Hugo Urquina, Agustín Petroni, Sandra Baez, Vladimir Lopez, Micaela do Nascimento, Eduar Herrera, Raphael Guex, Esteban Hurtado, Alejandro Blenkmann, Leandro Beltrachini, Carlos Gelormini, Mariano Sigman, Alicia Lischinsky, Teresa Torralva, Fernando Torrente, Marcelo Cetkovich, Facundo Manes

**Affiliations:** 1 Laboratory of Experimental Psychology and Neuroscience, Institute of Cognitive Neurology and Institute of Neuroscience, Favaloro University, Buenos Aires, Argentina; 2 National Scientific and Technical Research Council, Buenos Aires, Argentina; 3 Universidad Diego Portales, Santiago, Chile; 4 Integrative Neuroscience Laboratory, Physics Department, University of Buenos Aires, Buenos Aires, Argentina; 5 Pontificia Universidad Católica de Chile, Santiago, Chile; 6 Epilepsy Center, University of Buenos Aires – National Scientific and Technical Research Council – University of Buenos Aires, Buenos Aires, Argentina; 7 Laboratory of Industrial Electronics, Control and Instrumentation, National University of La Plata, La Plata, Argentina; 8 Universidad Autónoma del Caribe, Barranquilla, Colombia; 9 Pontificia Universidad Católica Argentina, Buenos Aires, Argentina; 10 Grupo de Investigación Neurored, Universidad Surcolombiana, Neiva, Colombia; 11 Laboratory for Behavioral Neurology and Imaging of Cognition, University of Geneva, Geneva, Switzerland; University of Adelaide, Australia

## Abstract

**Background:**

Adults with bipolar disorder (BD) have cognitive impairments that affect face processing and social cognition. However, it remains unknown whether these deficits in euthymic BD have impaired brain markers of emotional processing.

**Methodology/Principal Findings:**

We recruited twenty six participants, 13 controls subjects with an equal number of euthymic BD participants. We used an event-related potential (ERP) assessment of a dual valence task (DVT), in which faces (angry and happy), words (pleasant and unpleasant), and face-word simultaneous combinations are presented to test the effects of the stimulus type (face vs word) and valence (positive vs. negative). All participants received clinical, neuropsychological and social cognition evaluations. ERP analysis revealed that both groups showed N170 modulation of stimulus type effects (face > word). BD patients exhibited reduced and enhanced N170 to facial and semantic valence, respectively. The neural source estimation of N170 was a posterior section of the fusiform gyrus (FG), including the face fusiform area (FFA). Neural generators of N170 for faces (FG and FFA) were reduced in BD. In these patients, N170 modulation was associated with social cognition (theory of mind).

**Conclusions/Significance:**

This is the first report of euthymic BD exhibiting abnormal N170 emotional discrimination associated with theory of mind impairments.

## Introduction

Bipolar disorder (BD) is a neuropsychiatric disease that is characterized by unpredictable manic, hypomanic and depressive episodes. Recent studies of BD have reported social cognition deficits and emotional impairments [1], [2] with subtle abnormalities during the euthymic stages. Moreover, social cognition task based on emotional process [3], [4] are affected in BD [5]. For instance, emotional processing triggered by pictures [6] or faces [7], [8] is impaired in BD. Furthermore, facial emotional processing deficits have been associated with psychosocial impairments and mania risk in children and adolescents [9], [10]. Also, related disorders such anxiety and major depression [11], as well as schizophrenia [12], [13] presents impaired attribution of emotion to facial expressions. Indeed, brain networks involved in facial encoding and emotional processing overlap with the fronto-striatal circuit affected in pediatric-adolescent BD [14], [15]. Similarly, impaired facial emotion processing and social cognition seems to be related to connectivity deficits between the amygdala and temporal association cortical regions [16]. Nevertheless, the relationship between brain measures of face emotional processing and social cognition in adult euthymic BD is unknown.

Event-related potentials (ERPs) provide excellent temporal resolution of cognitive processing; this non-invasive method allows assessment of brain function and determination of factors that modulate it [17]. Specifically, the N170 is an ERP linked to face processing, with neural generators in the fusiform gyrus [18]. The N170 amplitude is primarily modulated by stimulus type discrimination (object recognition) of faces compared with objects or words [19], [20]. In addition, the N170 amplitude can be also modulated by emotional processing [21]–[23]. Thus, this component is an ideal brain marker to assess possible cortical markers of face structural processing and emotional modulation during the euthymic stage, an expected subtle impaired BD condition.

To our knowledge, only one study has investigated N170 modulation during emotional processing in BD. Degabriele et al specifically looked at ERPs in depressed BD type I patients using a paradigm of emotional inhibition on face presentation to modify the classic emotional go/no-go test [24]. The results showed abnormal P100 emotional processing and reduced N170 in BD patients. However, N170 reports are available only for depressed bipolar patients, but not patients in other states. Examining euthymic patients may provide evidence that face or emotional processing is affected irrespective of condition. No study of structural and emotional face-word processing and their relation to social cognition profile in euthymic BD has been reported.

Social cognition impairments are important markers of several disorders with abnormal frontotemporal activation [25]. The theory of mind (ToM hereafter) consists in the ability to attribute cognitive and affective mental states to one and others. Usually, the mental states of others are inferred using information from facial clues. Beliefs, intentions and other mental states are more reliably inferred when the emotion from face can be identified [26]. Since BD presents deficits in ToM (e.g., [5]) we expected to find an association between cortical processing of emotional faces and ToM.

We used an ERP design of a dual valence task [3], [22], [27], [28] in which faces and words were presented to test the effects of object recognition though stimulus type (ST: faces, words or simultaneous face-word) and valence (positive vs. negative). We recorded the N170 modulation of object recognition and emotional processing. We estimated the neural sources of this component (the main source of N170 seems to be the fusiform gyrus, and abnormalities in BD in this area have been already reported: [29], [30]). Finally, to assess the relationship between emotional processing and social cognition (theory of mind, ToM hereafter), a correlation analysis was used to compare the ERPs and the performance on two ToM tasks. We expected N170 deficits on face valence in faces, word and simultaneous stimuli in the BD participants. Moreover, N170 processing deficits of valence in BD would be related to social cognition performance (ToM).

## Materials and Methods

### Participants

Thirteen adult participants with BD (M = 40.1 years old, SD = 2.5 years, five females, twelve participants with right-handedness), and 13 healthy controls (M = 39.30 years old, SD = 2.51 years, five females, twelve participants with right-handedness) completed a full clinical, neuropsychological end electrophysiological evaluation. A questionnaire was given to controls to rule out hearing, visual, psychiatric or neurological deficits. Only participants with no such deficits were included.

### Ethics

All participants provided written informed consent (as outlined in the PLoS consent form) in agreement with the Helsinki declaration. Although some of the participants have diagnosis of bipolar conditions, any of those disorders implied a reduced capacity to consent. The Ethics Committee of the Institute of Cognitive Neurology approved this study. All data was analyzed anonymously.

### Patient Criteria and Recruitment Process

All participants fulfilled DSM-IV criteria for BD and were euthymic bipolar patients type II without any comorbidity. Diagnoses were made by three experts (AL, FT and MC). Absence of other disorders (eg, axis-II disorders, substance use disorders) was determined with the neuropsychiatric interview. The Beck Depression Inventory II (BDI-II; [31]) and the Young Mania Rating Scale (YMRS [32]) were used to evaluate depression and mania, respectively. To obtain scores of impulsivity and anxiety, the Barratt Impulsivity Scale (BIS11 [33]), and the State-Trait Anxiety Index (STAI [34]) were considered. To exclude possible comorbid attention deficit hyperactivity disorder (ADHD), we used the ADHD Rating Scale questionnaire for adults and children (Barkley and Murphy [35]). All patients were euthymic [scores ≤8 on the Montgomery-Asberg Depression Rating Scale (MDRS) and ≤6 on the YMRS] for at least 8 weeks and with no change in medication type or dosage over 4 months. Patients were not receiving antipsychotic medication.

### Neuropsychological Assessment

We assessed a neuropsychological evaluation in order to provide a profile of the patients regarding basic domains. Memory was evaluated using the Rey Verbal Learning Test (RVLT [36]). Attention and concentration were assessed using the Trail Making Test A (TMT-A [37]). Semantic fluency were measured using the Controlled Oral Word Association test (COWAT, [36]). An arithmetic test, Wechsler Adult Intelligence Scale III (WAIS III [38]) was also included.

Several tests were compiled to evaluate executive functioning. The INECO Frontal Screening test developed by our team (IFS, [39]) was used to assess frontal lobe function indexed by several subtasks: Motor Programming, Conflicting Instructions, Verbal Inhibitory Control, Abstraction, Backwards Digit Span, Spatial Working Memory, and Go/No Go. Digit Forward Span and TMT-B [37] were used to measure attentional flexibility, attentional speed, sequencing and planning skills. Numbers Key and Searching Symbols [38] were used to evaluate visual perception and organization, visual scanning and the efficient production of multiple motor responses. Phonological fluency was measured with the COWAT [36]. Finally, Ordering Letters and Numbers (Letters & Numbers) was used to assess mental manipulation and working memory [38].

### Social Cognition

Two social cognition tasks related to ToM were assessed. First, we included the Faux Pas test (FPT [40]), which evaluates hurtful or insulting social situations. Second, the Reading the Mind in The Eyes (RMTE, [41]), was used to assess emotional inference.

### Dual Valence Task (DVT)

The DVT [3], [22], [27], [28] is a paradigm based on previous ERP reports of the implicit association test [20], [42]. The DVT is a two-alternative forced-choice task displayed on a computer screen. Participants were asked to classify faces (angry vs happy), words (pleasant vs unpleasant), or simultaneous face-word stimuli according to their valence (positive or negative) as quickly as possible. Eighty happy and angry facial expressions and 142 pleasant and unpleasant word stimuli were included. The happy and angry sets of pictures depicted the same people. For training blocks (see below) different facial and semantic stimuli was used (see [22]). Faces were previously controlled for arousal, valence, emotion (angry vs happy) and physical properties; and words were controlled for arousal, valence, predictability, content, length and frequency (for details see [22]).

**Figure 1:**
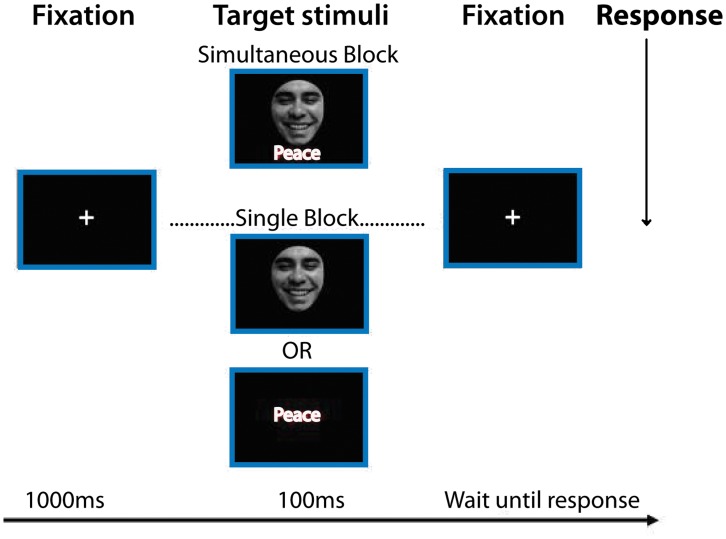
DVT. The trial starts with a fixation cross (1000 ms), followed by a target stimulus (100 ms): single stimulus face or word, (Single Stimulus Block) or simultaneously presented face and word (Simultaneous Stimuli Block). Finally a fixation cross was presented until a participant´s response was performed.

A trial began with a fixation cross for 1000 ms, and each stimulus was subsequently presented for 100 ms followed by a fixation cross until participant response. After a response, there was an interstimulus interval (ISI) of 1000 ms (see figure 1). Each stimulus was centered horizontally and vertically on the screen subtending a visual angle of 4.5°×3.15° at a viewing distance of approximately 80 cm. The DVT comprises 2 blocks (*Single stimulus block* and *Simultaneous stimuli block*) of 640 trials. The block sequence (first simultaneous followed by single vs first single followed by simultaneous) were counterbalanced across participants.

#### Single stimulus block

For each trial, participants were shown a face or a word in the center of the screen and responded as to whether the stimulus was angry or happy (faces), or pleasant or unpleasant (words). Single stimulus block trials were presented one by one with strict alternation between words and faces.

#### Simultaneous stimuli block

For each trial, participants were simultaneously shown a face in the center of the screen and a word 4 degree below the face. Participants responded as to whether the face was angry or happy, ignoring the word that was presented below.

### ERP Recordings

Electroencephalography (EEG) signals were sampled at 500 Hz with a Biosemi 128-channel system. The data were band-pass filtered (0.1 to 100 Hz) during recording and (0.3 to 30 Hz) off-line to remove unwanted frequency components [43]. During recordings the reference was set by default to link mastoids and re-referenced off-line to average electrodes. Two bipolar derivations monitored vertical and horizontal ocular movements (EOG). EEG data were segmented from 200 ms prior to 800 ms after the stimulus onset. All segments with eye movement contamination were removed from further analysis using ICA and a visual procedure. All conditions presented a trial rejection rate of <22%; and no differences between the groups with respect to ERP trial rejection rates were observed in any condition.

#### Source localization

In order to tests the involvement of specific areas of face processing (e.g., fusiform gyrus), distributed source models (8000 dipoles) of the N170 component for each condition were estimated using the standardized low-resolution brain electromagnetic tomography algorithm (sLORETA [44]). An average head model was used [45], including white matter anisotropy co-registered with the ICBM model [which provides spatial coordinates with the Montreal Neurological Institute (MNI) atlas]. The forward problem was solved using a finite element method that was constrained for location. Selected sources were tested for further analysis using the Friedman Test. Unlike the parametric repeated measures ANOVA or paired t-test, this test makes no assumptions about the distribution of the data [43]. Details of source analysis are providing in (Information S1, *source analysis details*).

### Data Analysis

Off-line processing and EEG data analyses were performed using Matlab software, the EEGLab toolbox and T-BESP software. To analyze scalp topography of the ERP components, regions of interest (ROIs) were chosen after visual inspection of each component. These regions were consistent with previous DVT ROI selection [22], [27], [28]. Each N170 ROI (left and right) included four adjacent electrodes of the canonical locations around T8 and T7 [46]: the N170 ROIs were the Biosemi channels A9, A10, A11 and A12 for the left hemisphere and B6, B7, B8 and B9 for the right hemisphere (see Figure S1, *channel locations and selected electrodes*). Based on previous reports of the DVT [3], [22], [27], [28] we analyzed the mean amplitude values. N170 latency is a very useful measure for some paradigms (e.g., assessing face inversion, face learning) but does not seem to be a good fit for our design. Several studies have shown that N170 amplitude but not latency is affected by the stimulus or valence type (e.g. [3], [19], [22], [27], [28]). We visually selected the 160–210 ms time window for N170 for mean amplitude ERP analysis. Because preliminary analyses revealed no differences between groups for P100, this component was not included in the report. We focused on the mean amplitude analysis of N170 (and not in P200) since the former is the most validated ERP measure of face processing (see Information S2, *Focus on N170 mean amplitude*).

Accuracy and N170 mean amplitudes were averaged for faces, words and simultaneous stimuli separately and analyzed with repeated measures ANOVA with the *stimulus type* (ST: faces, words, simultaneous) and *valence* (positive vs. negative) as within-subject factors. ERP data included the additional factor *hemisphere* (left and right locations) for consideration. In addition, a between-subject factor *group* (BD vs. controls) was included for all data sets. Pot hoc tests (Tukey HSD) were performed for pairwise comparisons.

Global scores were calculated for ERPs to perform correlations with ToM tasks: (a) Stimulus discrimination (face-minus-word) was calculated for N170 mean amplitude; and (b) valence discrimination scores (positive-minus-negative) were calculated for N170 mean amplitude (separately for faces, words and simultaneous stimuli). To test whether ERP measures of stimulus type and valence were associated with social cognition, global scores were correlated with the FPT and the RMET using Spearman’s rank correlations corrected for multiple comparisons [false discovery rate (FDR) correction, which controls the fraction of rejections that are false positives].

## Results

### Demographic and Clinical Assessment

Table 1 shows the overall results from the demographic, clinical, and neuropsychological assessments. No significant differences in age (F(1, 24) = 0.056, p = 0.81), gender (X^2^(1) = 0.15, p = 0.69), educational level (F(1, 24) = 0.26, p = 0.61), or handedness (X^2^(1) = 0.00, p = 1) were observed between groups.

**Table 1:** Demographic information and neuropsychological test performance.

		BD (*n* = 13)	CTRLS (*n* = 13)	P
	Age (years)	40.15 (9.59)	39.30 (8.5)	N.S
***Demographics***	Gender (M : F)	5∶8	5∶8	N.S
	Education (years)	16.77 (3.24)	17.38 (2.84)	N.S
	Handedness (R:L)	12∶1	12∶1	N.S
	BIS-11	54.23 (22.3)	43.46 (13.7)	N.S
	BDI-II	8.07 (7.06)	6.38 (7.73)	N.S
***Clinical Profile***	YMRS	0.30 (0.85)	0.53 (1.45)	N.S
	STAI – State	23.76 (6.16)	14.15 (4.77)	**0.0001**
	– Trait	27.61 (6.23)	18.61 (5.19)	**0.0005**
	TMT-A	42.92 (13.74)	30.23 (6.83)	**0.006**
	Digit repetition	14.46 (3.90)	19.15 (4.31)	**0.007**
	RAVLT			
***General***	Immediate	51.30 (9.44)	53.61 (6.17)	N.S
***Neuropsychology***	DL	7.1 (2.5)	8.2 (2.0)	N.S
	Delayed	11.23 (3.08)	11.23 (2.61)	N.S
	Recognition	14.38 (1.04)	14.46 (1.39)	N.S
	Semantic Fluency	21.00 (6.64)	28.61 (6.13)	N.S
	Digits Forward	4.85 (1.07)	5.46 (1.39)	N.S
	TMT-B	82.61 (41.24)	68.92 (32.34)	N.S
***Executive***	LNST	12.30 (2.92)	12.76 (2.08)	N.S
***Functions***	IFS Total Score	25.23 (2.74)	26.76 (3.03)	N.S
	Phonological fluency	19.07 (1.85)	25.15 (1.85)	**0.029**
***Social Cognition***	Faux pas	17.84 (1.99)	19.84 (0.55)	**0.001**
	The Mind in the Eyes	25.30 (4.49)	27.61 (2.36)	N.S

All data were expressed as the mean (SD) with statistical test results in the right-hand column. Abbreviations. CTRLS: Controls; BIS- 11: Barratt Impulsiveness Scale; BDI-II: Beck Depression Inventory; YMRS: Young Mania Rating Scale; STAI: State-Trait Anxiety Index; TMT: Trail Making; RALVT: Rey Auditory Verbal gLearning Test; IFS: INECO Frontal Screening; and LNST: Letters and Numbers Task. The p-values are shown when significant differences were found. In all other cases N.S. denotes a non-significant difference.

Since patients were euthymic, no differences were observed between groups for clinical measures of impulsivity (F(1, 24) = 2.18, p = 0.15), depression (F(1, 24) = 0.33, p = 0.56), or mania (F(1, 24) = 0.24, p = 0.62). However, compared to controls, BD participants showed significant differences for the anxiety scale STAI-State (F(1, 24) = 19.86, p<0.001) and STAI-Trait (F(1, 24) = 16.03, p<0.01).

### Neuropsychological Assessment

Significant differences between groups were observed in attention and concentration assessed with the TMT-A (F(1,24) = 8.89, p = 0.006) and digits repetition (F(1,24) = 6.24, p = 0.007). No group differences regarding memory were observed for the RVLT immediate recall (F(1,24) = 0.38, p = 0.54), delayed recall (F(1,24) = 0.000, p = 1.0) or recognition phase (F(1,24) = 0.25, p = 0.87).

The groups did not differ on most tests of executive functioning including the IFS total score (F(1,24) = 1.85, p = 0.18), digits span (F(1,24) = 1.60, p = 0.21), TMT-B (F(1,24) = 0.88, p = 0.35) and letters and numbers task (F(1,24) = 0.21, p = 0.64). However, compared to controls, the BD group scored lower on the phonological fluency task (F(1,24) = 5.39, p = 0.02).

### Social Cognition

Performance on the FPT was significantly reduced in BD (F(1, 24) = 12.14, p<0.001), suggesting failures in high levels of TOM processing. For the RMTE, no differences between groups were observed (F(1, 24) = 2.68, p = 0.11).

### Behavioral Results (DVT)

Both groups showed good levels of accuracy on the DVT (92%, SD = 0.06 for BD and 94%, SD = 0.04 for control group, see Table 2). A significant *valence* effect on accuracy was found for positive faces compared to negative ones (F (1, 24) = 6.01, p = 0.02). No *valence* effects were observed for words (F (1, 24) = 0.87, p = 0.35) or simultaneous stimuli (F (1, 24) = 1.39 p = 0.25). In addition, a significant effect of *ST* was found (F (2, 48) = 3.48, p = 0.03), indicating that simultaneous stimuli elicited lower accuracy compared to face and word stimuli. No differences between groups were observed. No others effects or interactions were found.

**Table 2:** DVT performance for patients and controls (fractions).

	Face +	Face −	word +	Word −	Sim + +	Sim + −	Sim − −	Sim − +
BD (Mean)	0.95	0.93	0.95	0.94	0.94	0.91	0.90	0.89
BD (SD)	0.03	0.09	0.04	0.04	0.05	0.07	0.09	0.09
Controls (Mean)	0.95	0.95	0.94	0.95	0.96	0.94	0.93	0.93
Controls (SD)	0.03	0.04	0.05	0.05	0.03	0.04	0.06	0.05

The signs + and – depict positive and negative emotional valences, respectively. The double signs in the last four columns indicate valence for faces and words, respectively.

### ERPs (DVT)

**Figure 2:**
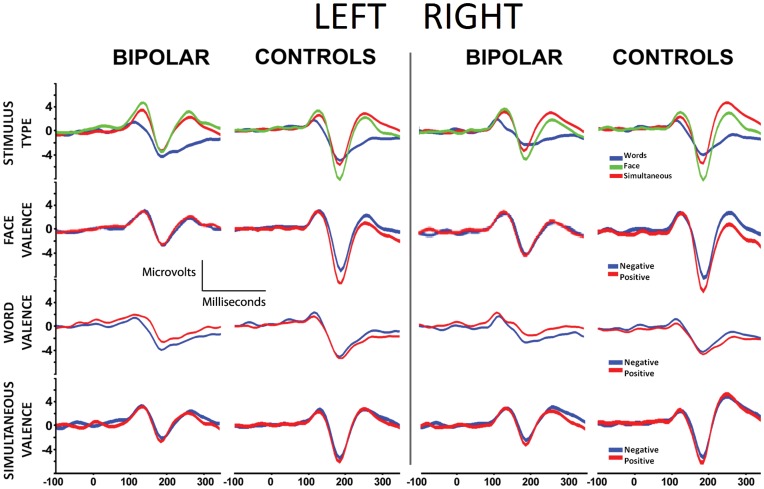
Main N170 results in the left and right hemisphere for both groups. First row: stimulus type effects (face vs word vs simultaneous). Second row: face valence effects (positive vs negative). Third row: word valence effects (positive vs negative). Fourth row: simultaneous valence effects (positive vs negative).

Figure 2 summarizes the ERP results and Table 3 shows the means ±SD for these conditions.

**Table 3:** N170 amplitude values in response to stimulus type and valence effects (Face, Word, and Simultaneous) for each group and hemisphere.

	BD		Controls	
	mean (SD)		mean (SD)	
	Left	Right	Left	Right
a. Stimulus Type (ST) Effects				
Faces	−0.65 (1.98)	−1.71 (1.31)	−5.57 (0.78)	−5.42 (0.98)
Words	−2.15 (1.30)	−1.06 (1.65)	−3.83 (0.90)	−3.23 (1.70)
Simultaneous	−0.88 (1.08)	−1.17 (1.58)	−3.86 (1.13)	−3.97 (1.90)
**b. Face Valence Effects**				
Positive	−0.31 (2.76)	−1.56 (1.50)	−6.94 (1.19)	−7.09 (1.45)
Negative	−1.00 (2.27)	−1.85 (1.38)	−4.20 (1.67)	−3.75 (1.02)
**c. Word Valence Effects**				
Positive	−1.28 (1.64)	−0.39 (1.62)	−3.90 (1.75)	−3.36 (1.91)
Negative	−3.02 (1.20)	−1.72 (1.58)	−3.75 (1.12)	−3.09 (1.10)
**d. Simultaneous Valence Effects**				
Positive	−1.2 (1.16)	−1.55 (1.22)	−4.20 (1.57)	−4.46 (1.24)
Negative	−0.55 (1.43)	−0.79 (1.63)	−3.51 (1.93)	−3.49 (1.00)

### Stimulus Type Effects

No main effects of *stimulus type* (*ST*; F(2, 48) = 1.74, p = 0.18) were observed. Furthermore, no effects of *hemisphere* (F (1, 24) = 0.02, p = 0.87) or interactions between *hemisphere*×*group* (F (1, 24) = 0.12, p = 0.73) were observed. Nevertheless, ST×*hemisphere* showed a significant interaction (F (2, 48) = 4.03, p = 0.02), indicating enhanced right lateralized effects of faces.

Individual group comparisons of ST suggested a similar pattern, with a preponderance of faces over other stimuli (both hemispheres in controls and the right hemisphere in BD; see Information S3, *Individual ST comparison in BD participants and controls*). Figure 2 (first row) shows the stimulus type modulation of ERPs for both groups, and Table 3 details all of the descriptive statistics.

### Face Valence

Main effects of *valence* (positive>negative: F (1, 24) = 4.16, p = 0.05) and *group* (controls>BD: F(1, 24) = 9.86, p = 0.004) were observed. No effects of *hemisphere* were found (F (1, 24) = 0.58, p = 0.45). An interaction of *group*×*valence* (F (1, 24) = 6.77, p = 0.02) was found. Post hoc analysis of this interaction (Tukey’s HSD test, MS = 17.69, df = 24.00) showed that positive valence faces elicited larger N170 amplitudes compared to negative valence faces in controls (p = 0.02). In contrast, BD patients did not show valence N170 modulation (p = 0.29) (see figure 2, second row).

A significant interaction between *valence*×*hemisphere* (F (1. 24) = 3.99, p = 0.05) evidenced emotional discrimination of facial stimuli in the right hemisphere. Moreover, a face *valence*×*group*×*hemisphere* interaction (F (1, 24) = 7.96, p = 0.009) indicated different lateralized group effects. A post hoc analysis of this interaction (Tukey’s HSD, MS = 14.79 df = 33.51) shown that N170 amplitudes in both hemispheres failed to discriminate face valence in BD (p>0.05). Conversely, N170 from controls were modulated in both hemispheres (p<0.005). In summary, BD patients exhibited significant deficits in N170 discrimination for positive face valence in the right hemisphere.

### Word Valence

For word stimuli, no effects of *valence* (F (1, 24) = 3.07, p = 0.09), *group* (F (1.24) = 2.79, p = 0.10), or *hemisphere* (F (1, 24) = 2.54, p = 0.12) were observed. Similarly, there were no interactions of *hemisphere*×*group* (F (1, 24) = 0.21, p = 0.64) or *valence*×*hemisphere* (F (1, 24) = 0.26, p = 0.60). However, a *group*×*valence* interaction was observed (F (1, 24) = 5.62, p = 0.03). In addition, enhanced N170 amplitude for negative words compared to positive ones was observed in BD patients (p<0.01), and the amplitude of negative valence words in BD patients was significantly enhanced compared to controls (p<0.05). No other effects were observed. Therefore, compared to the control group, the N170 amplitude in BD patients presented an early semantic valence modulation with an enhancement in response to negative words (Figure 2, third row).

### Simultaneous Valence

Faces presented simultaneously with interfering words yield a similar effect of face stimuli, evidencing *a m*ain effects of *group* (controls>BD; F (1, 24) = 6.46, p = 0.01) and *valence* (positive >negative; F (1, 24) = 4.33, p = 0.04). No effect of *hemisphere* (F (1, 24) = 0.14, p = 0.70) was detected. An interaction of *group*×*valence* (F (1, 24) = 5.23, p = 0.03) indicated different valence processing of simultaneous stimuli in both groups. Post hoc comparison of *group*×*valence* (Tukey’s HSD test, MS = 3.56, df = 24.00) showed that N170 from controls discriminated the face valence of simultaneous stimuli (positive>negative; p = 0.04), whereas BD patients did not (p>0.05). No other effects or interactions were significant (figure 2, fourth row).

### Source Analysis

**Figure 3:**
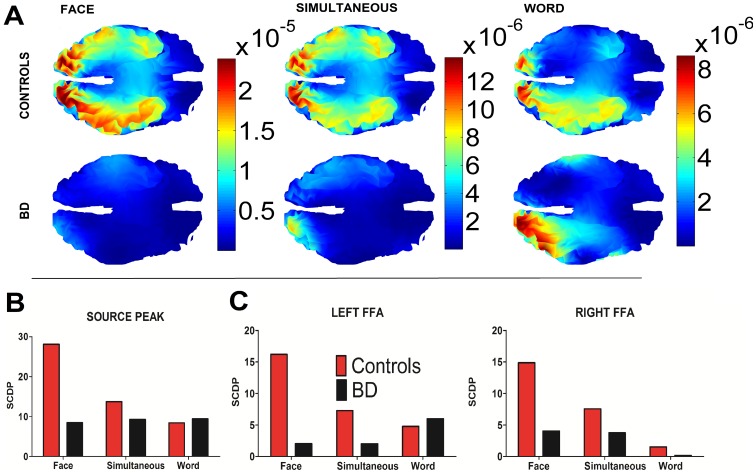
Cortical standardized current density power mapping of N170 (face, word, and simultaneous stimuli). A). N170 source imaging estimation of face, simultaneous and word stimuli for controls (top) and BD patients (bottom). B). Average values of estimated standardized current density power at maximum peaks of activation for each condition at the N170 window. C) Average values of N170 estimated standardized current density power at left and right face fusiform area (FFA) in the temporo-occipital fusiform gyrus. Non-parametric Friedman analysis revealed that FFA sources in controls showed decreasing activation from face to word stimuli (F = 4.66, p = 0.01). This effect was not observed in BD (F = 1.65, p = 0.19). The BD group exhibited reduced activation of FFA from faces compared to controls in both hemispheres [left: F = 3.31, p = 0.03; right: F = 5.86, p = 0.01)]. Word stimuli elicited strong activation of the left FFA in BD compared to controls (F = 6.22, p = 0.01). SCDP: standardized current density power.

Figure 3A depicts the activation distribution evoked by the three stimulus types (face, word and simultaneous), and figure 3B shows the mean source peak and the mean face fusiform area (FFA) intensity in both controls and BD participants. The source peak of N170 neural activity was observed in different regions of the fusiform gyrus (FG, details of spatial coordinates are provided in Table 4): left hemisphere for words, bilateral for faces and bilateral (rightpreponderant) for simultaneous stimuli. Standardized current density power (SCDP) was higher for faces and simultaneous stimuli but lower for words (in the left hemisphere) in controls than in patients, which is consistent with additional N170 group differences (see below). Following statistical comparisons of non-parametric analysis (see figure legend), for the FFA (figure 3B), controls showed decreasing activation from face to word stimuli. This effect was not observed in BD. The BD group exhibited reduced activation of FFA from faces compared to controls in both hemispheres (left MNI coordinates of ICBM: −32 −53 −18; right MNI coordinates of ICBM: 35 −48 −19). In addition, word stimuli elicited strong activation of the left FFA in BD compared to controls (see figure legend for statistical results).

**Table 4:** Estimation of N170 neural generators.

	Faces						
	Source peak					Left FFA	Right FFA
	MNI Coordinates			Anatomical Description (HOCSA)	SCDP	SCDP	
	X	Y	Z		Mean	Mean	Mean
Control	−18	−78	−17	32% Occipital Fusiform Gyrus; 19% Lingual Gyrus.	28.07	16.20	14.87
BD	−21	−93	−8	28% Left Occipital Pole, 5%; Occipital Fusiform Gyrus, 5% Lateral Occipital Cortex, inferior division.	8.49	2.01	4.02
	Simultaneous						
	Source peak					Left FFA	Right FFA
	MNI Coordinates			Anatomical Description (HOCSA)	SCDP	SCDP	
	X	Y	Z		Mean	Mean	Mean
**Control**	19	−85	−18	33% Right Occipital Fusiform Gyrus, 8% Lingual Gyrus, 4% Occipital Pole; 3% Lateral Occipital Cortex, inferior division.	13.71	7.29	7.56
**BD**	−24	−86	−20	35% Left Occipital Fusiform Gyrus, 8% Lateral Occipital Cortex, inferior division, 3% Occipital Pole, 3% Lingual Gyrus.	9.30	1.99	3.76
	**Words**						
	**Source peak**					**Left FFA**	**Right FFA**
	**MNI Coordinates**			**Anatomical Description (HOCSA)**	**SCDP**	**SCDP**	
	**X**	**Y**	**Z**		**Mean**	**Mean**	**Mean**
**Control**	−11	−85	−18	25% Left Occipital Fusiform Gyrus, 22% Left Lingual Gyrus.	8.42	4.78	1.52
**BD**	−13	−89	−19	33% Left Occipital Fusiform Gyrus, 10% Lingual Gyrus, 5% Occipital Pole 2% Left Lateral Occipital Cortex, inferior division.	9.46	5.96	0.17

Abbreviations. FFA: Face fusiform area in the temporo-occipital fusiform gyrus; HOCSA: Harvard-Oxford Cortical Structural Atlas; SCDP: standardized current density power. MNI coordinates for FFA: left (−32 −53 −18) and right (35 −48 −19). MNI coordinates were provided by ICBM.

In summary (figure 2 and Table 3), modulation of N170 by stimulus type was observed in both groups but BD patients showed N170 deficits in emotional modulation of facial and simultaneous stimuli. Furthermore, only BD patients showed an early discrimination in N170 amplitude for word valence in the left hemisphere, triggered by amplitude enhancement of negative stimuli.

### Correlation Analysis

We performed a correlation analysis between N170 scores (stimulus type and valence) and ToM tasks in both groups. In BD, N170 stimulus type discrimination demonstrated a positive correlation with FPT (r = 0.55 p = 0.05). Word valence also positively correlated with FPT (r = 0.61 p = 0.03) and RMTE (r = 0.56 p<0.05). In controls, face valence showed a correlation with RMET (r = 0.63 p = 0.01).

## Discussion

The primary goal of this study was to investigate cortical markers of facial and semantic emotion processing in euthymic BD and controls matched for gender, handedness, educational level and age. The secondary goal was to assess individual variability of social cognition (ToM) profiles related to N170.

In summary, our results suggest abnormal valence modulation in BD, associated with individual profiles of ToM. Compared to controls, BD patients presented reduced N170 and facial emotional modulation (for both facial and simultaneous stimuli). Furthermore, BD patients displayed early cortical discrimination of negative words valence, suggesting the activation of negative bias in the semantic stimuli processing. N170 source analysis of facial stimuli evidenced fusiform gyrus activation, which was reduced in affected participants. The two groups showed slightly dissimilar association of N170 with ToM processing. Previous reports of abnormal ERP responses [47] relating to configural processing of faces (fusiform gyrus), and mental state decoding (medial prefrontal lobe) in autism suggest a relationship between facial processing and normal social-cognitive skills [48]. To our knowledge, this is the first study that suggests abnormal N170 associated with ToM in euthymic BD.

From a clinical perspective, an important factor in BD is the vulnerability to stress and the stress-induced episodes. Stress has been proposed as an important neurobiological factor in BD [49]. Adequate emotional recognition and related ToM processing are required for emotional self-regulation. In this vein, abnormal processing of negative semantic information and face emotion would cause patients to have inappropriate stressful reactions to daily life events, even if these events are ambiguous or irrelevant.

### Behavioral Performance (DVT)

Because both groups presented high rates of accuracy in the DVT, inattention or task comprehension problems can be ruled out. It is likely that behavioral differences between groups were not found in this study because the BD participants were euthymic and not depressed or maniac, suggesting that impaired emotional processing in euthymic patients can be more subtle detected at an electrophysiological level. In fact, reports of ERP changes without overt behavioral differences in the DVT paradigm have been reported in other psychiatric comorbid populations such as adults with ADHD [27] and schizophrenia families [28]. This finding reveals that physiological responses may evidence subclinical aspects of early facial discrimination that may not necessarily reach consciousness or manifest explicitly through behavior.

### N170 Results

Our findings confirm a previously reported effect of stimulus type [19], [46] and valence [23] in controls. We replicated the left side preponderance for words; however, unlike earlier studies, we did not find a right lateralization for faces [23]. Rather, we found a bilateral effect for faces, which was in accordance with fMRI [50] and other ERP data [51]. Moreover, the combined pattern of valence and stimulus type replicates similar work employing the DVT [3], [22], [28] and their association with ToM [3].

BD patients exhibited a reduced N170 and abnormal emotional processing in the current study, confirming reports using other methodologies [7], [8], [52]. These results are also consistent with evidence of abnormal functional connectivity during face processing in children with BD [9]. Moreover, previous reports employing the DVT in schizophrenic families [28] and adults with ADHD [27] have evidenced similar N170 impairments. Taken together, these findings suggest that impaired emotional processing indexed by N170 may be a useful biomarker of potentially common genetic deficits. This assertion is also supported by the fact that related disorders, such as adults with anxiety disorders or major depression both have a deficit in recognizing facial expression of emotions [11].

Regarding word processing, the BD group showed amplitude enhancement of N170 negative valence for words in the left hemisphere, suggesting an enhanced early attentional bias toward negative information [53], [54]. This finding is consistent with the increased perception of BD to attentional allocation of negative rather than to positive emotional cues [16]. Although some studies in non-euthymic BD (manic) have shown positive bias [55], others have demonstrated attentional biases toward negative valence stimuli in depressed BD patients employing non-facial emotion processing tasks [56]. Furthermore, enhanced recognition of negative facial expressions has been reported in euthymic BD [7], [57]. The negative bias is compatible with reports of enhanced negative emotion processing in anxiety [58], and depression, triggered by a pessimistic attitude [59], [60]. Moreover, preserved frontotemporal networks are required in order to process negative emotions [61].

Only a previous N170 study has suggested that BD patients have abnormal cortical processing of facial emotional information [24] and has claimed a positive bias (enhanced P100 amplitudes to happy faces) and a decrease in N170 amplitude. Although both N170 studies on BD provide evidence of reduced N170 and cortical abnormalities in emotional processing, there are important differences. Several factors would explain the differences between those results and the present report:

- Degabriele et al [24] have used a go/no-go task with happy and fearful faces. This task did not elicit behavioral differences in emotional processing or N170 in controls. They only found emotional processing differences between groups in the P100, which is primarily modulated by attention. Consecuently, these results can be explained by attentional involvement (rather than emotional processing), as previously reported in BD [62]. The DVT considers faces (happy and angry) and words (positive and negative) and produces behavioral and N170 differences in object recognition and facial emotional processing in control participants [3], [22], [27], [28]. No between-group differences were observed for P100, suggesting that attentional processes do not explain the observed emotional differences.
- Degabriele et al [24] did not manipulate object recognition and thus did not provide a baseline for face processing. Stimulus type differences are a robust modulator of the N170 [19], [46]. Because Degabriele et al only used faces, their results do not provide a marker of object recognition or information about the specificity of the observed emotional impairments. Moreover, the exclusive use of facial stimuli precludes N170 habituation effects [63]. Possible habituation effects are a confounding factor regarding the absent N170 emotional modulation observed in both controls and patients.
- Different BD groups were present in both studies. Degabriele et al recruited depressed BD type I but did not formally assess mania. They included patients on antipsychotic medications that affect electrophysiological signals [64]. Finally, controls had significantly different educational levels. Our study compared euthymic bipolar type II disorder with controls that were matched for age, gender, handedness and educational level. No patients currently taking antipsychotic medications were included.
- Finally, we included an N170 source analysis and an association measure of N170 and social cognition (see following section for this last point). These revealed that, consistent with previous reports, the main source of the N170 was located in the FG, including the FFA for faces [3], [18], [27] and in the left FG for words [3], [19], [63].

Theoretical models of emotional face perception [4] propose parallel and interactive systems indexing object recognition (triggered by the FG) and emotional discrimination (triggered by the amygdala). In BD patients, basic and structural face integration processes (e.g., stimulus type effects) seem to be relatively preserved, whereas more subtle processes, such as emotional processing, seem to be altered (reduced for faces and enhanced for words) at early stages of N170 processing (see [65]). This finding may be due to reduced connectivity between the amygdala and the FG, consistent with neuroimaging studies of FG abnormalities in BD [29], [30].

### Limitations

This study is the first to associate N170 impairment to ToM in euthymic BD adults. Our study was comprised of a relatively small number of BD patients because we excluded those with comorbidities or antipsychotic medication use. However, our sample size is comparable to previous reports of BD assessed with electrophysiology (e.g., euthymic BD [66], [67]) or behavioral measures [68], [69]. Future studies should evaluate the multivariate association between basic cortical processes and complex clinical, cognitive and social profiles in a larger sample. As with almost all previous studies, BD patients in the current study were taking medications (other than antipsychotics). Therefore, we cannot discount the influence of these drugs on cognitive function. Finally, a longitudinal study could identify factors of comorbidity, course and recovery in these BD. Nevertheless, there are considerable aspects that maximize the power of this study. Namely, the clear operational definitions and stringent inclusion criteria, together with the control for affective symptoms, and accurate matching of patients with healthy controls according to age, gender, handedness and educational level ensure proper characterization of each group.

### Neurocognitive of BD and their Relation to Early N170

As expected given their euthymic condition, patients presented a profile comparable to controls. In agreement with previous BD reports, we found higher levels of anxiety [70], subtle attentional deficits [71] and some failures in executive tasks [72], [73]. In addition, as previously demonstrated [5], we found lower performance in BD group on FPT, suggesting failures in TOM processing.

The social-perceptual component of TOM consists of the ability to perceive mental states of others based on observable information, such as facial expressions, and the capacity to distinguish between faces and objects [74]. Accordingly, our results show stimulus type and word valence associated with TOM measures, which may be related with the deficits observed in BD. Thus, early abnormal semantic and face processing would influence object recognition and emotional categorization processes, affecting the building blocks of social cognition skills. Healthy subjects showed an attentional bias for negative emotions in the unconscious condition while this valence bias remained absent in MDD patients. The automatic negative bias in BD, consistent with the already reported negative bias in depression may have important implications for clinical diagnosis and therapy.

Previous reports on BD [75] stress the relationship between impairments in social cognition and associated dysfunction of emotional regulation [76] in BD [9]. By integrating N170 research and social cognition our report contributes to the understanding of the neural basis and social cognition profile in bipolar disorder.

## Supporting Information

Figure S1 **Channel locations and selected electrodes.** Figure shows the overall ERP response to faces in the DVT and the ellipses contain selected electrodes for left (A8 to A12) and right N170 (B6 to B9).
(TIF)

Information S1 **Source analysis details.**
(DOCX)

Information S2 **Focus on N170 mean amplitude.**
(DOCX)

Information S3 **Individual ST comparison in BD participants and controls.**
(DOC)
