# Supplementary figures and images for: Neural Processing of Emotional Facial and Semantic Expressions in Euthymic Bipolar Disorder (BD) and Its Association with Theory of Mind (ToM)
Source: PLoS One. 2012 Oct 8;7(10):e46877. doi: 10.1371/journal.pone.0046877 (PMC3466207; doi:10.1371/journal.pone.0046877)

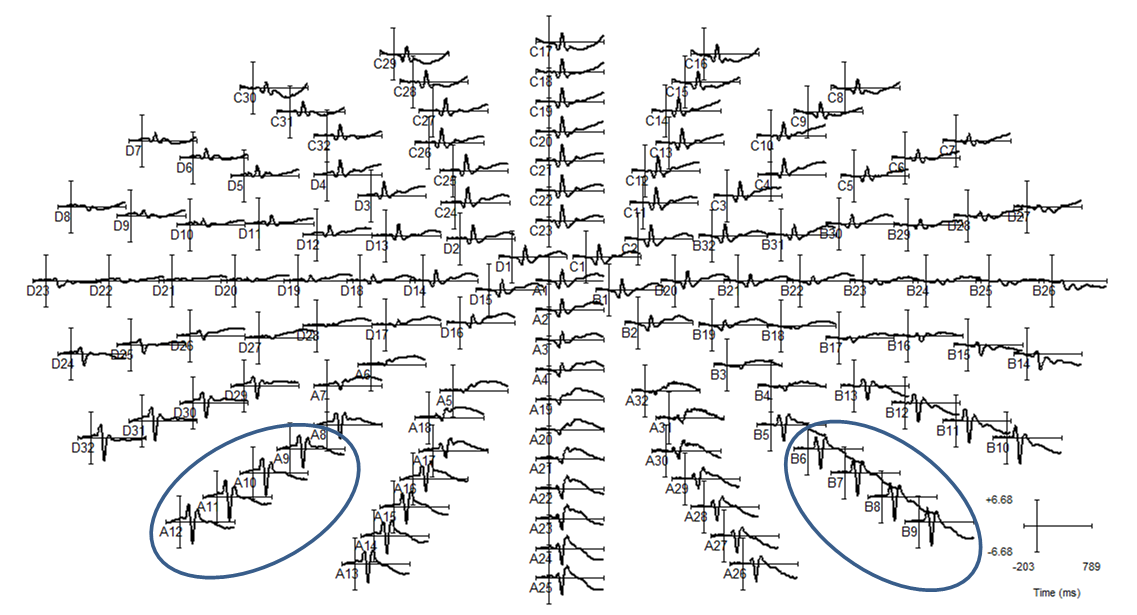

Supplement: Figure S1 — Channel locations and selected electrodes. Figure shows the overall ERP response to faces in the DVT and the ellipses contain selected electrodes for left (A8 to A12) and right N170 (B6 to B9). (TIF) [file pone.0046877.s001.tif]
