# Supplementary material for: Neural Processing of Emotional Facial and Semantic Expressions in Euthymic Bipolar Disorder (BD) and Its Association with Theory of Mind (ToM)
Source: PLoS One. 2012 Oct 8;7(10):e46877. doi: 10.1371/journal.pone.0046877 (PMC3466207; doi:10.1371/journal.pone.0046877)
Supplement: Information S1 — Source analysis details. (DOCX) [file pone.0046877.s002.docx]

**S1. Source analysis details**

Distributed source models (8000 dipoles) were estimated using the standardized low resolution brain electromagnetic tomography algorithm (sLORETA; Pascual-Marqui, 2002). These locations were derived by performing a location-wise inverse weighting of data, with a minimum norm least squares analysis of their estimated variances, leading to a smooth solution. An average head model built from a sample of 152 MRIs provided by the International Consortium of Brain Mapping (ICBM) was used (Mazziotta et al., 2001). To make a more realistic model we considered white matter anisotropy by using a diffusion tensor average atlas of 81 healthy participants co-registered with the ICBM model (which provides spatial coordinates with the MNI atlas from the Montreal Neurological Institute). The forward problem was solved using the finite element method (Zhang et al., 2004).

Possible solutions were constrained for location to the cortical surface but were not constrained for orientation. This head model is useful for source localization when individual MRI data are not available. As previously reported with source analysis of dual valence task (DVT) (Ibanez et al., 2011), and since temporal differences occur between stimulus types, the local minimum within the N170 window was considered for each of these. The average of ERP for faces, simultaneous and words stimuli were obtained for each group. Average signal for the N170 representative electrodes was calculated, within a 150-210 ms time window for faces and simultaneous stimuli, and within a 160-230 ms time window for word stimuli for each group. N170 peak amplitude was found as the local minimum of this average. Potentials of all channels at local minimum were extracted for each condition. Finally, standardized current density power (SCDP) was obtained for each condition and group.

Ibáñez, A., Petroni, A., Urquina, H., Torrente, F., Torralva, T., Hurtado, E., Guex, R., Blenkmann, A., Beltrachini, L., Muravchik, C., Baez, S., Cetkovich, M., Sigman, M., Lischinsky, A., Manes, F., 2011. Cortical deficits of emotional face processing in adults with ADHD: Its relation to social cognition and executive function. Social Neuroscience

Mazziotta, J., Toga, A., Evans, A., Fox, P., Lancaster, J., Zilles, K., Woods, R., Paus, T., Simpson, G., Pike, B., Holmes, C., Collins, L., Thompson, P., MacDonald, D., Iacoboni, M., Schormann, T., Amunts, K., Palomero-Gallagher, N., Geyer, S., Parsons, L., Narr, K., Kabani, N., Le, G.G., Boomsma, D., Cannon, T., Kawashima, R., Mazoyer, B., 2001. A probabilistic atlas and reference system for the human brain: International Consortium for Brain Mapping (ICBM). Philos. Trans. R. Soc. Lond B Biol. Sci. 356, 1293-1322.

Pascual-Marqui, R.D., 2002. Standardized low-resolution brain electromagnetic tomography (sLORETA): technical details. Methods Find. Exp. Clin. Pharmacol. 24 Suppl D, 5-12.

Zhang, L., Gerstenberger, A., Wang, X., Liu, W.K., 2004. Immersed finite element method. Computer Methods in Applied Mechanics and Engineering. 193, 2051-2067.
