# Supplementary material for: Neural Processing of Emotional Facial and Semantic Expressions in Euthymic Bipolar Disorder (BD) and Its Association with Theory of Mind (ToM)
Source: PLoS One. 2012 Oct 8;7(10):e46877. doi: 10.1371/journal.pone.0046877 (PMC3466207; doi:10.1371/journal.pone.0046877)
Supplement: Information S2 — Focus on N170 mean amplitude. (DOCX) [file pone.0046877.s003.docx]

**S2. Focus on N170 mean amplitude**

We focused only in N170 (and not in P100 and P200) for several reasons. Preliminary analysis reveals no groups differences on P100. The N170 is the formal ERP assessing the early stage of facial processing. The P100 reflects earliest sensory processing stage, and its emotional modulation would be triggered by attentional factors (Hillyard et al., 1998) and not necessarily by a facial emotional process. Contrarily, N170 is a well characterized component of early face processing with neural sources related to face-specific areas. The same cannot be said for P200.

This component seems to have different functional properties than N170 and their relation with N170 is yet to be established. In addition, all previous studies reporting N170 from paradigms including faces and words (e.g., Ibañez et al., 2010, 2011a, 2011b, 2011c; Zhu et al., 2010) had not reported P200. To our knowledge, the N170 is the best marker of early face processing. Finally, the N170 (and not the P100 or P200) has reported as being related to executive functions and social cognition (Petroni et al., 2012). Consequently, we focused our analysis in the N170 component.

Hillyard, S.A., Teder-Salejarvi, W.A., Munte, T.F., 1998. Temporal dynamics of early perceptual processing. Curr. Opin. Neurobiol. 8, 202-210.

Ibanez, A., Gleichgerrcht, E., Hurtado, E., Gonzalez, R., Haye, A., Manes, F.F., 2010. Early Neural Markers of Implicit Attitudes: N170 Modulated by Intergroup and Evaluative Contexts in IAT. Front Hum. Neurosci. 4, 188.

Ibanez, A., Hurtado, E., Riveros, R., Urquina, H., Cardona, J.F., Petroni, A., Lobos-Infante, A., Barutta, J., Baez, S., Manes, F., 2011a. Facial and semantic emotional interference: a pilot study on the behavioral and cortical responses to the Dual Valence Association Task. Behav. Brain Funct. 7, 8.

Ibanez A; Riveros R; Hurtado E; Gleichgerrcht E; Urquina H; Herrera, E; Amoruso, L Martin-Reyes M; Manes F. 2011b. The face and its emotion: Cortical Deficits in Structural Processing and Early Emotional Discrimination in Schizophrenic and Relatives. Psychiatry Research, DOI10.1016/j.psychres.2011.07.027

Ibáñez, A., Petroni, A., Urquina, H., Torrente, F., Torralva, T., Hurtado, E., Guex, R., Blenkmann, A., Beltrachini, L., Muravchik, C., Baez, S., Cetkovich, M., Sigman, M., Lischinsky, A., Manes, F., 2011c, In press. Cortical deficits of emotional face processing in adults with ADHD: Its relation to social cognition and executive function. Social Neuroscience

Petroni, A., Urquina, H., Guex, R., Hurtado, E., Manes, F., Sigman, M., Ibáñez, A. (2012). Early cortical measures of valence, stimulus type discrimination and interference: association to executive function and social cognition. Neuroscience Letters

Zhu, X.R., Zhang, H.J., Wu, T.T., Luo, W.B., Luo, Y.J., 2010. Emotional conﬂict occurs at an early stage: evidence from the emotional face–word Stroop task. Neuroscience Letters. 478, 1–4.
