# Supplementary material for: Neural Processing of Emotional Facial and Semantic Expressions in Euthymic Bipolar Disorder (BD) and Its Association with Theory of Mind (ToM)
Source: PLoS One. 2012 Oct 8;7(10):e46877. doi: 10.1371/journal.pone.0046877 (PMC3466207; doi:10.1371/journal.pone.0046877)
Supplement: Information S3 — Individual ST comparison in BD participants and controls. (DOC) [file pone.0046877.s004.doc]

**S3. Individual ST comparison in BD participants and controls**

In controls, no effects for hemisphere (F (1. 25) = 0.33, p= 0.56) or interactions between ST and hemisphere (F (2. 50) = 1.75, p = 0.18) were found. A significant stimulus type effect (F (2. 50) = 10.41, p=0.0001) was observed. Compared to words, post hoc comparisons over this interaction showed N170 amplitude enhancement in both hemispheres for faces (left p<0.001; right p<0.001) and simultaneous stimuli (left p<0.01; right p<0.005) respectively.

BD group not shown significant effects of stimulus type (F (2.50) = 1.63 p= 0.20). However, the stimulus type x hemisphere showed a significant interaction (F (2.50) = 3.90, p = 0.02). Post hoc comparisons performed over this interaction (Tuckey HSD, df MS = 1.51, df = 50.00) showed enhanced of N170 amplitude in the left hemisphere (faces ˃ simultaneous stimulus; p = 0.01) and similar results compared faces and words in the right hemisphere (p<0.002).
